# Examination of the Position Accuracy of Implant Abutments Reproduced by Intra-Oral Optical Impression

**DOI:** 10.1371/journal.pone.0164048

**Published:** 2016-10-05

**Authors:** Hitoshi Ajioka, Hidemichi Kihara, Chikayuki Odaira, Takuya Kobayashi, Hisatomo Kondo

**Affiliations:** Department of Prosthodontics and Oral Implantology, School of Dentistry, Iwate Medical University, Morioka, Iwate, Japan; University of North Carolina at Chapel Hill, UNITED STATES

## Abstract

An impression technique called optical impression using intraoral scanner has attracted attention in digital dentistry. This study aimed to evaluate the accuracy of the optical impression, comparing a virtual model reproduced by an intraoral scanner to a working cast made by conventional silicone impression technique. Two implants were placed on a master model. Working casts made of plaster were fabricated from the master model by silicone impression. The distance between the ball abutments and the angulation between the healing abutments of 5 mm and 7 mm height at master model were measured using Computer Numerical Control Coordinate Measuring Machine (CNCCMM) as control. Working casts were then measured using CNCCMM, and virtual models via stereo lithography data of master model were measured by a three-dimensional analyzing software. The distance between ball abutments of the master model was 9634.9 ± 1.2 μm. The mean values of trueness of the Lava COS and working casts were 64.5 μm and 22.5 μm, respectively, greater than that of control. The mean of precision values of the Lava COS and working casts were 15.6 μm and 13.5 μm, respectively. In the case of a 5-mm-height healing abutment, mean angulation error of the Lava COS was greater than that of the working cast, resulting in significant differences in trueness and precision. However, in the case of a 7-mm-height abutment, mean angulation errors of the Lava COS and the working cast were not significantly different in trueness and precision. Therefore, distance errors of the optical impression were slightly greater than those of conventional impression. Moreover, the trueness and precision of angulation error could be improved in the optical impression using longer healing abutments. In the near future, the development of information technology could enable improvement in the accuracy of the optical impression with intraoral scanners.

## Introduction

In recent years, the field of dentistry has developed through the advances in information technology, primarily computer-aided design/computer-aided manufacturing system (CAD/CAM) [1, 2]. The computer-aided systems brought novel treatment options responding to the diverse needs of the patient, particularly esthetics [3].

The optical intraoral impression system can reproduce shapes of cavities, abutment teeth, and adjacent teeth via visible ray. Takuma et al. have previously reported that optical impression techniques together with CAD/CAM might achieve acceptable fitting accuracy for the three-unit fixed partial denture [4], and also Nayyar et al. showed that those new technologies could fabricate procedures and methods for the implant prosthesis [5]. In addition, optical three-dimensional (3D) imaging systems were more efficient techniques than conventional impressions. [6]. CEREC^®^ system enables to fabricate a ceramic restoration in a day, thereby saving time and reducing the load on both dentists and patients [7–10]. Moreover, CAD/CAM technology can simplify treatment procedures and reduce time for appointments, but careful acquisition of data with precise execution is essential to achieve success [11]. Kattadiyil et al. succeeded in the fabrication of a removable partial denture framework using an intraoral scanner to capture soft tissue precisely in the case of a limited tooth-supported clinical situation [3]. Converting digital information on the state of the oral cavity makes it possible to accumulate, process, and transmit data [12]. Examples include the simplification of the replication and reproduction of prosthesis, efficiency of work by additional processing and removal processing [13], and the order of the prosthesis to remote facilities by data transfer on wireless network [5]. In addition, digital data measured using an intraoral scanner is predicted to facilitate combining cone-beam computed tomography [14, 15], tracer, articulator, and dental spectrophotometer [16] using an open system.

The current standard methods for dental implant impressions need vinyl polysiloxane (VPS), an individual-tray and impression copings. Despite the deformation of impression materials and the expansion of dental casts, conventional impressions for implants prosthesis have proved successful results in clinical practice [17].

A newly established procedure in which all manufacturing processes are handled in digital data is termed the *digital workflow* [18]. A digital workflow requires neither conventional plaster nor silicone impressions materials for the manufacturing process of the prosthesis [19, 20]. Consequently, dimensional changes in the physical properties are reduced, and the digital workflow should provide expected permanent dimensional stability [21, 22]. Moreover, no concerns regarding a flawed impression of conventional technique remain because the optical impression can perform additive scan and stitches the images with additional scanning allowing the clinician to identify any deficiencies [3]. If required, acquired 3D data (stereo lithography, STL, format) can produce a highly accurate STL model, using 3D printer [23, 24]. On the other hand, the optical impression could be useful when patients have difficulties for conventional impression because of a vomiting reflex, pathologic tooth mobility, and lockjaw.

Currently, the optical impression has used in implant treatment. The STL data that was obtained from optical impressions has been used to design custom abutment and superstructure [25–27]. So there are many literatures that described accuracy of STL data. There were two evaluating methods of STL data models: One method measured between two points of experimental group and control group using STL data [28] and the other method was that whole model images was superimposed by computer automatically (best fit algorithm) [21, 29–31]. However, these studies has been using STL data as a control. It is considered that STL data certainly cause an error of about 5 micrometers or more.

Although several reports have described fitting accuracy of crown or bridge made with the optical impression technique, few manuscripts concerning fitting accuracy of implant prosthesis have been published [25, 32, 33]. The purpose of this study was to evaluate accuracy of the optical impression using CNCCMM as a control, comparing virtual models reproduced by an intraoral scanner to models casted by conventional silicone impression. And the control was measured by CNCCMM. Because CNCCMM is in error by less than one micrometer.

## Materials and Methods

### Fabrication of master model, working casts, and virtual model

**Fig 1:**
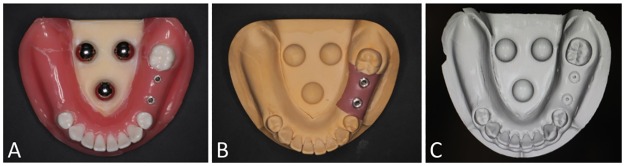
(A) Master model. (B) Working cast produced by conventional impressions. (C) Virtual model converted after the optical impression.

Three calibration spheres (φ10.0 mm steel ball: a grade 28 (JIS B 1501, ISO 3290), Sato Tekkou, Japan) made of metal were fixed to a lower jaw model which missed the second premolar and the first molar (Implant Practice Jaw Model: NISSIN, Japan) **using** self-curing acrylic resin (UnifastIII: GC, Tokyo, Japan). Subsequently, two implants with an external hex connection system (MkIII groovy RP φ4.0 × 10.0 mm: Novel Biocare, Switzerland) were placed on the second premolar and first molar regions as shown in Fig 1A.

Open type custom trays were made of room temperature curing resin(OSTRONII: GC, Japan), lying 1.4 mm paraffin wax(PARAFFIN WAX: GC, Japan), to produce working casts from master model. Conventional impression was carried out on the basis of the general clinical procedures. Impression copings connected to the implants on the master model were tightened by 15 Ncm of torque using a torque wrench (prosthetic torque wrench: Nobel Biocare, Switzerland). VPS tray adhesive (Vinyl Polysiloxane Tray Adhesive: 3M Espe, Saint Paul, USA) was applied to the open trays before the impression. Ten conventional impressions were made with VPS impression material (Imprint 3 light- and heavy-body impression material: 3M Espe) and the open tray. The tray was removed with less resistance. The impressions were removed after 5 min and the implant replicas were connected to the impression copings. To assume an actual working cast, silicone (Gi-Mask: Coltene Whaledent, Switzerland) was applied around the cervical part of the implant replicas to simulate peri-implant soft tissue before pouring plaster (GC New Fujirock: GC, Japan). Plaster was poured into all impressions within 2 h (Fig 1B).

The master model was scanned with an intraoral scanner (Lava COS: 3M Espe, software version 3.0.2) after dusting with titanium oxide powder (Lava Powder: 3M Espe), and the scan data were exported as STL data. The following measurements were performed subjecting to STL data obtained from the intraoral scanner by 3D analyzing software (Focus Inspection: Nikon, Japan) (Fig 1C).

### Measurements

**Fig 2:**
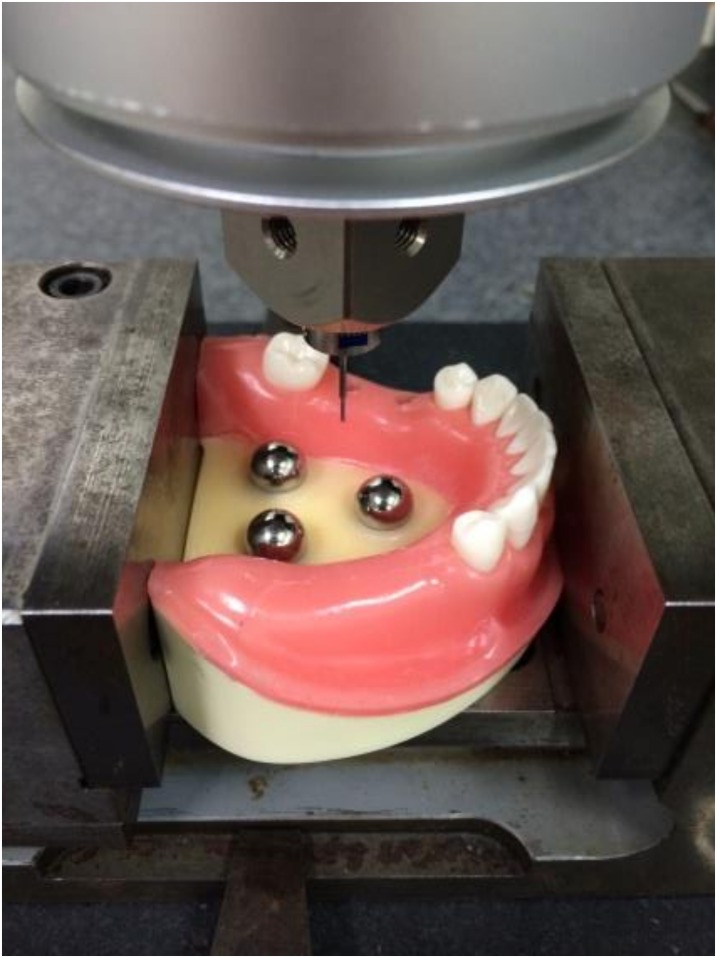
Measurement of master model with CNCCMM. CNCCMM was used to examine the master model to obtain the virtual true value regarding the 3D positions of the implants.

The master model was measured with an industrial Computer Numerical Control Coordinate Measuring Machine (CNCCMM) (UPMC 550-CARAT: Curl Zeiss, Germany) to obtain control value of the 3D positions of the implants (Fig 2). The accuracy of CNCCMM was certified by a maximum permissible error for length measurement of 0.8 + L/600 μm (L: measuring length, mm) according to ISO 10360–2.

**Fig 3:**
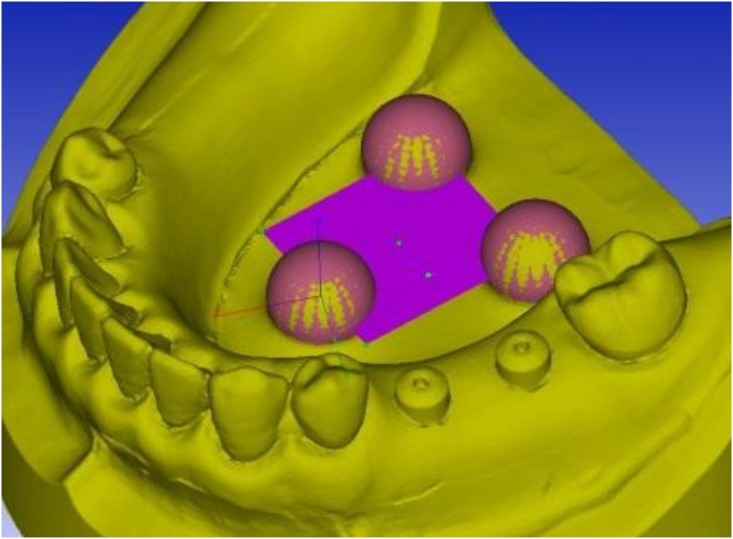
Setting of reference plane and reference point on STL data.

As a control, master model was scanned 10 times with CNCCMM at Iwate Industrial Research Institute. To evaluate the error of intraoral scanner, the master model was scanned 10 times with the intraoral scanner. To evaluate the error of conventional impression, ten working casts were scanned with CNCCMM. Those processes were performed at 20°C ± 0.5°C room temperature and 50% ± 10% humidity. The three reference spheres incorporated into the master model and the working casts were used to create a horizontal reference plane and a reference point (Fig 3).

### Evaluation of accuracy

**Fig 4:**
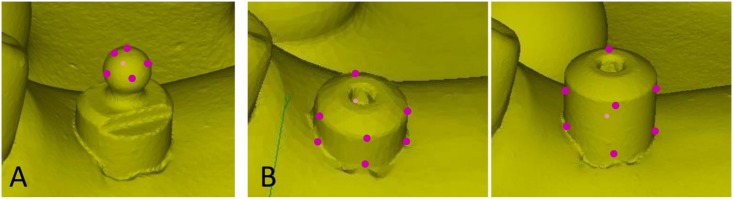
Extraction of the center point and center line. (A) Six points on the surface of the ball abutment were plotted to define the center point of the ball abutments. (B) Eight points on the surface of the 5-mm- or 7-mm-height healing abutments were plotted to define the central axis of the abutments representing the central axis of implants.

The distance was measured between to ball abutments (Brånemark System RP φ5.0 mm: Nobel Biocare, Switzerland) both in the master model and the working casts. The ball abutments were connected to the implants and tightened by 15 Ncm of torque using a torque wrench and a driver (machines driver: Nobel Biocare, Switzerland). The center of the ball abutment was identified by the detection of six points on the ball abutment, and the distance between the center points of the ball abutments was calculated by the software (Focus inspection) (Fig 4A).

The center point of the ball abutment on the second premolar implant was defined as PA: (***x***_***A***_, ***y***_***A***_, ***z***_***A***_), and the one on the first molar implant was defined as PB: (***x***_***B***_, ***y***_***B***_, ***z***_***B***_). Distance between PA and PB was defined as D that was calculated with the following formula:

$$D=\sqrt{{\left(x_{A}-x_{B}\right)}^{2}+{\left(y_{A}-y_{B}\right)}^{2}+{\left(z_{A}-z_{B}\right)}^{2}}$$

Angulation between the two implants was measured with two implants. A guide pin (guide pin implant level Brånemark System RP 20 mm: Nobel Biocare, Switzerland) was used to measure the angulation as control; 5-mm- or 7-mm-height healing abutments (healing abutment Brånemark System RP φ5.0 × 5.0 mm: RP φ5.0 × 7.0 mm: Nobel Biocare, Switzerland) were tightened by 15 Ncm of torque. The angle between the centerlines of the 5-mm- or 7-mm-height healing abutments was calculated by divide the x-, y-, and z-components of the center of the direction vector. The peripheries were considered cylinders plotted in eight points (Fig 4B).

The centerline of the healing abutment on the second premolar implant was defined as LA: (***i***_***A***_, ***j***_***A***_, ***k***_***A***_), and the centerline of the healing abutment on the first molar implant was defined as LB (***i***_***B***_, ***j***_***B***_, ***k***_***B***_). Angulation between LA and LB was defined as **θ** that was calculated from the following formula:

$$\theta ={\text{cos}}^{-1}\left(i_{A}i_{B}+j_{A}j_{B}+k_{A}k_{B}\right)$$

### Statistics

The F-test was conducted for each impression technique and each item by the t-test and Wilcoxon signed-rank test, and the level of statistical significance was set at *P* < 0.05.

## Results

### Distance error between the ball abutments connected to implants

**Fig 5:**
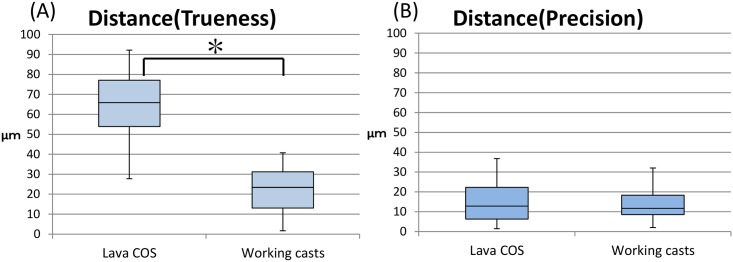
Boxplot of accuracy measurement in distance error between two ball abutments (trueness and precision). (A) Distance error of trueness: STL data by Lava COS is significantly different from working casts made from conventional impression (*P* < 0.05). (B) Distance error of precision: STL data by Lava COS is not significantly different from working casts made from conventional impression (*P* < 0.05).

The distance of two ball abutments on the master model measured using CNCCMM ranged from 9633.3 μm to 9636.6 μm and mean distance was 9634.9 ± 1.2 μm (95% confidence interval: 9634.0–9635.7 μm). The trueness of distance error of the master model as measured by Lava COS ranged from 27.7 μm to 92.2 μm, and that of working casts as measured using CNCCMM ranged from 1.7 μm to 40.8 μm. The mean of distance error of the master model as measured by Lava COS was 64.5 ± 19.0 μm (95% confidence interval: 51.0–78.1 μm). The mean of distance error of the working casts as measured using CNCCMM was 22.5 ± 12.4 μm (95% confidence interval: 13.7 μm to 31.4 μm) (Fig 5A). The precision of distance error of the master model as measured by Lava COS ranged from 1.5 μm to 36.9 μm, and that of working casts as measured using CNCCMM ranged from 2.0 μm to 32.0 μm. The mean of distance error of the master model as measured by Lava COS was 15.6 ± 10.9 μm (95% confidence interval: 7.9 μm to 23.3 μm). The mean of distance error of the working casts as measured using CNCCMM was 13.5 ± 8.6 μm (95% confidence interval: 7.4 μm to 19.6 μm) (Fig 5B). The t-test resulted in a significant difference between Lava COS and working casts for trueness and precision. The distance resulted in a significant difference between Lava COS and working casts for trueness by t-test (*P* < 0.05). In contrast, the distance resulted in no significant difference between Lava COS and working casts for precision by t-test (*P* > 0.05).

### Angulation error between the healing abutments connected to the implants

The angulation of the master model as measured using CNCCMM ranged from 3.0854° to 3.1841°. The mean angulation was 3.1376 ± 0.0379° (95% confidence interval: 3.1104° to 3.1647°).

**Fig 6:**
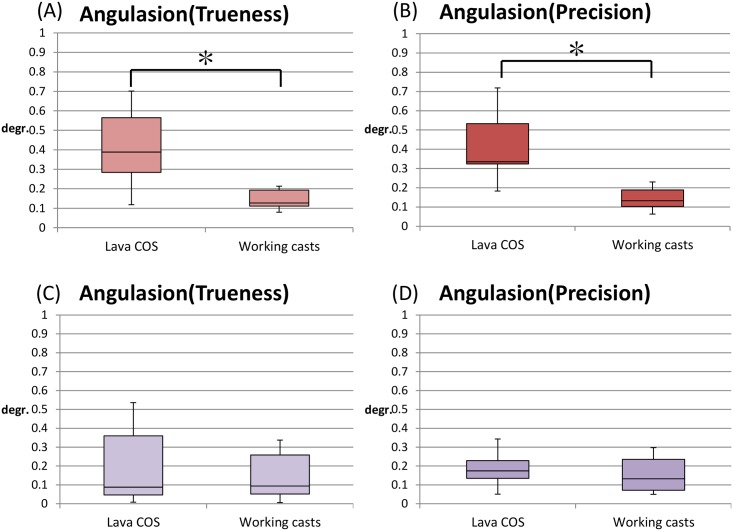
Box plot of trueness and precision of angulation error between two healing abutments with different height. The angulation between two healing abutments connected to the implants in the master model scanned by Lava COS was compared to those on the working casts as measured using CNCCMM for healing abutment of 5 mm and 7 mm height. (A) Angulation error of trueness: STL data by Lava COS is significantly higher than working casts made from conventional impression for the healing abutment of 5 mm height (*P* < 0.05). (B) Angulation error of precision: STL data by Lava COS is not significantly higher than working casts made from conventional impression for healing abutment of 5 mm height (*P* < 0.05). (C,D) Angulation error of trueness and precision: STL data by Lava COS is not significantly higher than that by working casts made from conventional impression for healing abutment of 7 mm height (P < 0.05).

The trueness of angulation error of the master model as measured by Lava COS ranged from 0.1185° to 0.7011°, and working casts as measured using CNCCMM ranged from 0.0798° to 0.2130° for a healing abutment of 5 mm height. The mean of angulation error of the master model as measured by Lava COS was 0.4154° ± 0.1781° (95% confidence interval: 0.2879°–0.5427°), and working casts as measured using CNCCMM was 0.1444° ± 0.0050° (95% confidence interval: 0.1798°–0.1798°) for a healing abutment of 5 mm height (Fig 6A). The precision of angulation error of the master model as measured by Lava COS ranged from 0.1830° to 0.7186°, and working casts as measured using CNCCMM ranged from 0.0632° to 0.2297° for a healing abutment of 5 mm height. The mean of angulation error of the master model as measured by Lava COS was 0.4154° ± 0.1661° (95% confidence interval: 0.2966°–0.5342°), and working casts as measured using CNCCMM was 0.1417° ± 0.0559° (95% confidence interval: 0.1011°–0.1811°) for a healing abutment of 5 mm height (Fig 6B).

The trueness of angulation error of the master model as measured by Lava COS ranged from 0.0083° to 0.5356°, and working casts as measured using CNCCMM ranged from 0.0058° to 0.3373° for a healing abutment of 7 mm height. The mean of angulation error of the master model as measured by Lava COS was 0.2032° ± 0.1989° (95% confidence interval: 0.0609°–0.3455°), and working casts as measured using CNCCMM was 0.1460° ± 0.1199° (95% confidence interval: 0.0602°–0.2317°) for a healing abutment of 7 mm height (Fig 6C). The precision of angulation error of the master model as measured by Lava COS ranged from 0.0511° to 0.3428°, and working casts as measured using CNCCMM ranged from 0.0498° to 0.2972° for a healing abutment of 7 mm height. The mean of angulation error of the master model as measured by Lava COS was 0.1871° ± 0.0931° (95% confidence interval: 0.1204°–0.2538°), and working casts as measured using CNCCMM was 0.1570° ± 0.0894° (95% confidence interval: 0.0931°–0.2209°) for healing abutment of 7 mm height (Fig 6D).

The healing abutments of 5 mm height resulted in a significant difference between Lava COS and working casts for trueness and precision by Wilcoxon signed-rank test (*P* > 0.05). On the other hand, healing abutments of 7 mm height resulted in no significant difference between Lava COS and working casts for trueness and precision by t-test (*P* > 0.05).

## Discussion

Recent reports described that slight errors occur during intraoral scanning for each specific scanning system [28]. CEREC AC projected a light stripe pattern on the object using a point-and-click system. iTero projected a parallel confocal laser on the object using a point-and-click system. In contrast, Lava COS used active wavefront sampling called as video image system. Andriessen et al. showed that the point-and-click system was less reliable in the case of small numbers of anatomical landmarks like the edentulous alveolar ridge [17]. Depending on the type of scanner, reflections on the surface of metal and the permeability of tooth may affect the acquired images. Therefore, dusting the surface with a titanium oxide powder is sometimes necessary before scanning.

Lee et al. reported that there were no significant differences between a pick-up impression and a transfer impression in the case of less than three implants, but many reports showed that pick-up impressions were more accurate in the case of more than four implants [34]. Some studies mentioned the accuracy of impressions was affected by the angulation of the implants. Two studies reported that the impression accuracy of an inclined implant was worse than that of a parallel implant, because of the deformation of impression material when the impression is removed [35, 36]. On the contrary, in an optical impression, there are no distortions in removal from the oral cavity and no cast deformations. Therefore, an optical impression should get a smaller error with respect to the angulation than a conventional impression would get. In this study, implants were substantially parallel, so there was no significant difference between conventional impression and optical impressions.

The scan body developed for optical impressions [17] and the digitally encoded healing abutment can virtually reproduce implant positional information for manufacturing individual custom abutments and superstructures [25–27]. Welnder et al. stated that avoiding removal of the healing abutment encourages peri-implant soft tissue stability and might inhibit peri-implant bone loss [37]. Particularly, Encode^™^ system was noted to reduce the number of abutment removals. In this study, the reference plane and reference points were obtained from the use of three precise metallic balls. Therefore, the landmark was reproducible, and it was possible to measure precisely between the implants.

Results of our study showed that the trueness of distance error was 64.5 μm for the Lava COS and 22.5 μm for the working casts. The distance error of working cast was smaller than that of Lava COS. Those errors could influence the compatibility of prosthesis. However, the precision of distance error was 15.6 μm for the Lava COS and 13.5 μm for the working casts. There were few variations between multiple measurements. Ender et al. reported that comparing Lava COS, CEREC, and conventional impressions, there were no significant differences in trueness [21]. However, in another study, digital impressions were not as true or precise as conventional impressions. Consequently, they suggested that digital impressions could not replace conventional impressions in restorative procedures temporarily [29].

In this study, angulation error was determined for both 5 mm and 7 mm height healing abutments. In case of 5-mm-height healing abutments, mean angulation error of the Lava COS was greater than the error of the working cast, suggesting that trueness and precision of the optical impression is inferior to conventional impression. However, in case of 7-mm-height healing abutments, the angulation error of Lava COS was not significantly different from the working cast in trueness and precision. Thus, the height of healing abutment may affect the error level for angulation in the Lava COS. In this study, analytical software might be able to recognize the actual shape of cylinders and precisely calculate the angulation when longer healing abutments were applied.

The results obtained in our study suggest that error of the optical impression was greater than that of conventional impression. However, the results might have proved only limited and temporal phenomenon because the trends in digital dentistry are drastic, and a number of newly developed apparatuses may be provided even today, suggesting that further analyses must be conducted continuously to acquire the esteemed information. In the near future, the development of information technology should enable improvement in the accuracy of the optical impression with intraoral scanners.

## Conclusions

In this study, distance error of the optical impression was slightly greater than that of conventional method. Using longer healing abutment, the trueness and precision of angulation error were improved in the optical impression.

## Supporting Information

S1 Table Detail 10 times data of accuracy measurement in distance error between two ball abutments (trueness and precision). (DOCX)

S2 Table Detail 10 times data of angulation error between two healing abutments of 5 mm height (trueness and precision). (DOCX)

S3 Table Detail 10 times data of angulation error between two healing abutments of 7 mm height (trueness and precision). (DOCX)
