# Supplementary material for: Examination of the Position Accuracy of Implant Abutments Reproduced by Intra-Oral Optical Impression
Source: PLoS One. 2016 Oct 5;11(10):e0164048. doi: 10.1371/journal.pone.0164048 (PMC5052018; doi:10.1371/journal.pone.0164048)
Supplement: S1 Table — (DOCX) [file pone.0164048.s001.docx]

S1 Table

Detail 10 times data of accuracy measurement in distance error between two ball abutments (trueness and precision).

|  | Ball abutment | | | | |
| --- | --- | --- | --- | --- | --- |
|  | trueness | |  | precision | |
|  | Lava COS | Working casts |  | Lava COS | Working casts |
| 1 | 63.05007 | 31.99506 |  | 1.485507 | 11.86981 |
| 2 | 86.71582 | 40.76702 |  | 22.18024 | 20.64178 |
| 3 | 77.68774 | 28.82972 |  | 13.15216 | 8.704476 |
| 4 | 42.2497 | 18.10232 |  | 22.28588 | 2.022925 |
| 5 | 92.19404 | 28.62864 |  | 27.65846 | 8.503394 |
| 6 | 27.72022 | 11.93565 |  | 36.81537 | 32.0609 |
| 7 | 68.69171 | 16.53633 |  | 4.156124 | 3.588912 |
| 8 | 75.33138 | 38.00083 |  | 10.7958 | 17.87559 |
| 9 | 59.71198 | 8.634358 |  | 4.823602 | 11.49089 |
| 10 | 52.00317 | 1.693818 |  | 12.53242 | 18.43143 |

(μm)
