# Supplementary material for: Examination of the Position Accuracy of Implant Abutments Reproduced by Intra-Oral Optical Impression
Source: PLoS One. 2016 Oct 5;11(10):e0164048. doi: 10.1371/journal.pone.0164048 (PMC5052018; doi:10.1371/journal.pone.0164048)
Supplement: S2 Table — (DOCX) [file pone.0164048.s002.docx]

S2 Table

Detail 10 times data of angulation error between two healing abutments of 5 mm height (trueness and precision).

|  | Ball abutment | | | | |
| --- | --- | --- | --- | --- | --- |
|  | trueness | |  | precision | |
|  | Lava COS | Working casts |  | Lava COS | Working casts |
| 1 | 0.315226 | 0.194757 |  | 0.250802 | 0.211392 |
| 2 | 0.267078 | 0.115218 |  | 0.331502 | 0.098583 |
| 3 | 0.273825 | 0.079828 |  | 0.338249 | 0.063193 |
| 4 | 0.654168 | 0.213035 |  | 0.718591 | 0.22967 |
| 5 | 0.701149 | 0.109449 |  | 0.636726 | 0.126084 |
| 6 | 0.118547 | 0.131941 |  | 0.18297 | 0.115306 |
| 7 | 0.605967 | 0.210736 |  | 0.541543 | 0.194101 |
| 8 | 0.388316 | 0.187379 |  | 0.323892 | 0.170744 |
| 9 | 0.388316 | 0.121562 |  | 0.323892 | 0.138197 |
| 10 | 0.441119 | 0.080051 |  | 0.505543 | 0.063416 |

(°)
