# Supplementary material for: Examination of the Position Accuracy of Implant Abutments Reproduced by Intra-Oral Optical Impression
Source: PLoS One. 2016 Oct 5;11(10):e0164048. doi: 10.1371/journal.pone.0164048 (PMC5052018; doi:10.1371/journal.pone.0164048)
Supplement: S3 Table — (DOCX) [file pone.0164048.s003.docx]

S3 Table

Detail 10 times data of angulation error between two healing abutments of 7 mm height (trueness and precision).

|  | Ball abutment | | | | |
| --- | --- | --- | --- | --- | --- |
|  | trueness | |  | precision | |
|  | Lava COS | Working casts |  | Lava COS | Working casts |
| 1 | 0.529298 | 0.263838 |  | 0.336457 | 0.208634 |
| 2 | 0.397888 | 0.070517 |  | 0.205047 | 0.125721 |
| 3 | 0.535601 | 0.105028 |  | 0.34276 | 0.049824 |
| 4 | 0.051738 | 0.337263 |  | 0.141103 | 0.282059 |
| 5 | 0.24398 | 0.045691 |  | 0.051139 | 0.100895 |
| 6 | 0.043645 | 0.005803 |  | 0.236486 | 0.061007 |
| 7 | 0.045358 | 0.082984 |  | 0.147483 | 0.138188 |
| 8 | 0.008345 | 0.242045 |  | 0.201186 | 0.297249 |
| 9 | 0.060264 | 0.299656 |  | 0.132577 | 0.244452 |
| 10 | 0.116272 | 0.006704 |  | 0.076569 | 0.061908 |

(°)
